# Childhood Fish Consumption and Learning and Behavioral Disorders

**DOI:** 10.3390/ijerph13111069

**Published:** 2016-11-02

**Authors:** Jenny L. Carwile, Lindsey J. Butler, Patricia A. Janulewicz, Michael R. Winter, Ann Aschengrau

**Affiliations:** 1 Department of Epidemiology, Boston University School of Public Health, Boston, MA 02118, USA; jennycarwile@gmail.com (J.L.C.); ljbutler@bu.edu (L.J.B.); 2 Department of Environmental Health, Boston University School of Public Health, Boston, MA 02118, USA; paj@bu.edu; 3 Data Coordinating Center, Boston University School of Public Health, Boston, MA 02118, USA; mwinter@bu.edu

**Keywords:** ADD, ADHD, fish, learning disorders, methylmercury

## Abstract

Fish is a major source of nutrients critical for brain development during early life. The importance of childhood fish consumption is supported by several studies reporting associations of n-3 polyunsaturated fatty acid (n-3 PUFA) supplementation with better behavior and school performance. However, fish may have a different effect than n-3 PUFA alone due to the neurotoxic effects of methylmercury, a frequent contaminant. We investigated associations of childhood fish consumption with learning and behavioral disorders in birth cohort study of the neurotoxic effects of early life exposure to solvent-contaminated drinking water. Childhood (age 7–12 years) fish consumption and learning and behavioral problems were reported in self-administered questionnaires (age 23–41 at questionnaire completion). Fish consumption was not meaningfully associated with repeating a grade, tutoring, attending summer school, special class placement, or low educational attainment. However, participants who ate fish several times a week had an elevated odds of Attention Deficit Disorder/Attention Deficit Hyperactivity Disorder (odds ratio: 5.2; 95% confidence interval: 1.5–18) compared to participants who did not eat fish. While these findings generally support the safety of the observed level of fish consumption, the absence of a beneficial effect may be attributed to insufficient fish intake or the choice of relatively low n-3 PUFA fish.

## 1. Introduction

Fish is the major dietary source of the long-chain n-3 polyunsaturated fatty acids (n-3 PUFA) eicosapentaenoic and docosahexaenoic acid (DHA) [1], nutrients critical for the development of cell membranes in the brain and retina, and subsequently, proper neural and visual function. Gestation represents a critical period for brain development, and, hence, the impact of maternal fish consumption on neurodevelopmental problems in children has been the subject of much research. Notably, children of women who regularly consumed fish, particularly low mercury varieties, during their pregnancies tend to score higher on developmental tests [2] and may also be protected against developing Attention Deficit Hyperactivity Disorder (ADHD)-related behaviors [3] relative to children of women with little or no prenatal fish consumption. When components of fish have been investigated individually, prenatal DHA exposure appears associated with improved cognitive development [4], while prenatal mercury exposure has been associated with ADHD symptomology [3,5] and, in some studies, lower estimated IQ [6].

Important brain development, particularly remodeling of the prefrontal cortex and synaptogenesis, however, continues throughout childhood, and fish intake during this period may also impact cognitive function. The importance of childhood fish consumption is supported by several studies, including randomized controlled trials, reporting associations of n-3 PUFA supplementation with better cognition, behavior, and school performance in healthy children [7], as well as improved symptoms in children diagnosed with Attention Deficit Disorder (ADD)/ADHD [8]. Few studies, however, have investigated fish consumption [9,10,11,12,13], which may have a different effect on learning and behavioral problems than n-3 PUFA alone due to the effects of other components of fish, particularly the neurotoxin methylmercury.

We hypothesized that overall childhood fish consumption would be associated with improved learning and fewer behavioral problems, but suspected that for typical consumers of high-mercury fish, the detrimental effects of methylmercury would outweigh any n-3 PUFA benefit, resulting in relatively worse outcomes. We investigated childhood fish consumption (overall, canned tuna, low-mercury, and high-mercury) and multiple learning and behavioral problems in a cross-sectional study of Cape Cod (MA, USA) residents.

## 2. Materials and Methods

### 2.1. Study Population

The Cape Cod Health Study is a retrospective cohort study of individuals born between 1969 and 1983 in one of eight Cape Cod, Massachusetts towns with documented vinyl lined (VL)/asbestos cement (AC) water pipes [14,15]. The cohort was originally designed to investigate associations of prenatal exposure to PCE (tetrachloroethylene or perchloroethylene), a VL/AC pipe leachate and drinking water contaminant, with multiple health outcomes [14,15,16]. Study participants additionally reported detailed information on their childhood fish intake.

We assessed whether an individual was eligible for the original study based on their residential proximity to VL/AC pipes [14]. Briefly, we selected “index” participants by comparing the maternal address listed on the birth certificate with information from a visual inspection of pipe distribution maps. Participants with a birth residence adjacent to a VL/AC pipe were initially considered exposed to PCE (N = 1910). We randomly selected a comparison group of participants unexposed to PCE who were frequency matched to PCE-exposed participants on month and year of birth (N = 1928). We additionally identified 1202 older siblings of the index participants who were also born in Massachusetts between 1969 and 1983. Between 2006 and 2008, we located the current address and telephone number of selected individuals, and sent them an introductory letter and self-administered questionnaire containing questions on childhood fish consumption and learning and behavioral problems. If an individual did not respond, we sent them three follow-up letters, and, if that was also unsuccessful, attempted to contact them by phone. Of the 5040 selected individuals, 332 could not be located, 111 were deceased, and 2908 refused to participate or failed to respond, leaving 1689 eligible individuals.

The majority of participants had a mother enrolled in a previous cohort, the Cape Cod Family Health Study [17,18,19]. Between 2002 and 2003, we used birth certificates and self-administered questionnaires to obtain data on the woman, her exposures during her pregnancy with the participant, and characteristics of the participants themselves. For this analysis, we excluded participants whose mother was not enrolled in the Cape Cod Family Health Study (N = 318) and participants with missing data on childhood fish consumption (N = 73). We additionally excluded participants who reported an exposure or condition that may have impaired their learning or behavior, including multiple birth (N = 35), prenatal exposure to ≥7 alcoholic drinks/week (N = 22) or known teratogenic medications (N = 8), and a history of deafness or blindness (N = 16), mental retardation (N = 4), or lead poisoning (N = 6). After further exclusion of those missing data on key covariates (N = 28), 1179 were considered eligible for the present analysis. Participants with missing outcome data were excluded from relevant analyses (N missing: ADD/ADHD, N = 12; tutoring for reading, N = 9; tutoring for math, N = 45; special class placement, N = 8; IEP, N = 11; summer school, N = 7; repeating a grade, N = 11; low educational attainment, N = 1). Enrollment rates were similar for participants who did and did not report childhood fish consumption (Table 1).

### 2.2. Assessment of Fish Consumption

Between 2006 and 2008, participants self-reported their fish consumption between the ages of 7 and 12 years. Fish was defined as “any kind of fish, including fish sticks and canned tuna fish”, but not including “seafood such as lobster, clams, scallops, shrimp, oysters, or any other kind of shellfish”. Shellfish were excluded because they are generally low in mercury [20]. Participants reported (a) how often they consumed fish (“did not eat fish”, “once a month or less”, “a couple of times a month”, “about once a week”, or “several times a week”) and (b) the type of fish they typically consumed. We additionally queried participants on whether or not they consumed specific varieties of fish (“did you eat any of the following kinds of fish?”), which were selected for their popularity (e.g., canned tuna and fish sticks) or high-mercury content (e.g., fresh tuna, swordfish, bluefish, and shark).

Using data on the type of fish each participant typically ate in conjunction with mean mercury concentrations published by the U.S. Food and Drug Administration (FDA) [20], we categorized participants reporting some fish consumption as “typical” consumers of one of the following: low-mercury fish, canned tuna, or high-mercury fish (Table S1). While mercury levels vary considerably among fish we considered to be high-mercury varieties (range: 0.32–1.00 μg·mercury/g), they each met or exceeded those of fish for which the FDA/Environmental Protection Agency (EPA) recommends limited or no consumption for pregnant women, nursing mothers, and young children. Specifically, the FDA/EPA limits consumption of canned albacore tuna (0.35 μg·mercury/g) to one meal weekly and advises complete avoidance of swordfish (1.00 μg·mercury/g) and other high-mercury varieties [20,21] for these populations. We considered canned tuna as a separate category because it was the most commonly reported variety of fish and has separate consumption guidelines. The specific variety of canned tuna (i.e., chunk light or albacore) was not obtained from participants making this category likely a heterogeneous combination of low- (i.e., chunk light) and high- (i.e., albacore) mercury fish. Typical consumers of low-mercury fish or canned tuna were considered “occasional” high-mercury fish consumers if they ever reported eating fresh tuna, swordfish, bluefish, or shark. Thus, each participant was assigned (a) an overall frequency of fish consumption; (b) a variety of fish typically consumed; and (c) a frequency of high-mercury fish consumption.

### 2.3. Assessment of Learning and Behavioral Problems

As part of the self-administered questionnaire administered between 2006–2008, participants were asked about whether a doctor or other health care provider ever diagnosed them with “Attention Deficit Disorder (ADD)/Attention Deficit Hyperactivity Disorder (ADHD)”, their year of diagnosis, whether they had ever taken medication for ADD/ADHD, and if they had “more difficulty paying attention or sitting still in school than most other children (their) age”. Participants also reported whether, between kindergarten and 12th grade, they ever received tutoring for reading; tutoring for math; were assigned to “a special class because of academic problems or behavioral problems”; had an Individualized Education Plan (IEP); attended summer school because of academic problems; or repeated a grade, as well as the grade of first occurrence of each outcome. Lastly, participants reported their highest level of education completed. We considered a high school degree or less to be low educational attainment.

We calculated Kappa statistics to assess the consistency of learning and behavioral problems self-reported by the participant with those recalled by his or her mother. All outcomes showed good (κ = 0.67 for ADD/ADHD, 0.68 for high school degree or less, and κ = 0.76 for repeating a grade) or fair (0.41 ≤ κ ≤ 0.58 for tutoring for reading, tutoring for math, special class placement, and IEP) agreement. We were unable to calculate consistency for summer school, which was not reported by the participants’ mothers. Accuracy of ADD/ADHD diagnosis recall was also supported by concurrent reports of associated symptoms and medication use (87% of participants reporting ADD/ADHD had difficulty paying attention or sitting still in school relative to their peers and 77% took medicine for ADD/ADHD).

### 2.4. Assessment of Covariates

For most index participants, we obtained data on date of birth, sex, birth weight and gestational duration, paternal occupation, maternal education, and their parents’ ages at the time of birth from birth certificates. For siblings of index participants and those with missing data, we obtained these data from self-report. Data on other covariates, including participant race, maternal alcohol use and smoking during the pregnancy, and family history of ADD/ADHD and other learning disabilities were collected as part of the self-reported questionnaire. Details on estimation of PCE exposure from contaminated drinking water have been previously described in detail [14].

### 2.5. Statistical Analysis

We calculated odds ratios (OR) and 95% confidence intervals (CI) for associations between fish consumption and learning and behavioral problems using logistic regression and, to account for sibling pairs, generalized estimating equations (GEE). First, we investigated the association between overall frequency of childhood fish consumption with each of eight individual learning and behavioral problems (ADD/ADHD, tutoring for reading, tutoring for math, attending summer school, special class placement, IEP, repeating a grade, and low educational attainment). To assess dose-response, we created a corresponding ordinal variable representing the number of fish meals per month, and modeled the exposure as a continuous variable. Second, we modeled the exposure as the type of fish typically consumed during childhood (i.e., low-mercury-fish, canned tuna, or high-mercury fish). Third, we modeled the exposure according to frequency of high-mercury fish consumption (i.e., ate fish, but not high-mercury fish; ate fish, occasionally high-mercury fish; ate fish, typically high-mercury fish). For each analysis, we considered participants who did not consume fish (N = 122) as the reference group.

Multivariable GEE models were adjusted for the following variables selected for their associations with ADD/ADHD [22,23,24] and educational problems [25,26]: maternal age (≤21, 22–25, 26–29, ≥30 years) and education at time of birth (high school diploma or less, some college, 4-year college degree or more), and participant race (white, other), sex (male, female), year of birth (1969–1974, 1975–1980, 1981–1983), and combined gestational age/birthweight (preterm or <2500 g, term and ≥2500 g). We also assessed the following potential confounders: maternal alcohol consumption or smoking during pregnancy, family history of ADD/ADHD or other learning disabilities, paternal occupation at the time of birth, and PCE exposure status (both pre- and postnatal, none). None had a meaningful impact on our findings when individually included in multivariate models and were therefore omitted from final adjusted models.

## 3. Results

### 3.1. Study Population

Of the 43% of participants (N = 451) who reported a learning or behavioral problem, 247 (48.6%) reported a single problem, 106 (N = 20.9%) reported two problems, and 155 (30.5%) reported three or more problems. With the exception of ADD/ADHD (median age at diagnosis = 16 years), outcomes first occurred during elementary school (median grade of occurrence: repeating a grade, grade 1; tutoring for reading, grade 2; special class placement and IEP; grade 3; tutoring for math, grade 4). The grade at which summer school was attended was not reported. Participants who reported consuming fish several times a week during childhood had a slightly later occurrence of ADD/ADHD diagnosis (median 18 years vs. 15 years) and repeating a grade (median grade 4 vs. grades 2) than non-fish consumers. The median grade at first occurrence was otherwise similar across categories of childhood fish consumption.

Outcomes were, in general, highly correlated. For example, among participants diagnosed with ADD/ADHD, 35% received tutoring for math, 26% received tutoring for reading, 33% had an IEP, 29% attended summer school, 45% were placed in a special class, 26% repeated a grade, and 28% had a high school degree or less.

### 3.2. Overall Frequency of Childhood Fish Consumption

The majority of participants (N = 1057; 90%) reported some childhood fish consumption. Participants who consumed fish most frequently were slightly older when they completed the questionnaire and more likely to be male, have a father employed in a blue-collar occupation, and a family history of learning disabilities and ADD/ADHD relative to participants with less frequent fish consumption (Table 2). Participants in every category of overall fish consumption were more likely to report an ADD/ADHD diagnosis compared to non-fish consumers, with those consuming fish several times a week having 4.5-times the odds of ADD/ADHD (95% confidence interval (CI): 1.2, 16) as non-fish consumers. Similar findings were observed following multivariable adjustment for predictors of learning and behavioral problems (for fish several times a week vs. no fish, odds ratio (OR): 5.2; 95% CI: 1.5, 18). Participants who consumed fish several times a week were also more likely to report that they had difficulty paying attention or sitting still compared to their peers than non-fish consumers (for fish several times a week vs. no fish, OR: 2.1, 95% CI: 1.1–3.9). Elevated, but generally non-statistically significant ORs were also observed for the association between frequency of fish consumption and repeating a grade (for fish consumption several times a week vs. no fish consumption, unadjusted OR: 1.6 (95% CI: 0.8–3.5); adjusted OR: 1.9 (95% CI: 0.8, 4.5)) (Table 3). More frequent childhood fish consumption was not associated with tutoring for reading or math, special class placement, IEP, attending summer school, or low educational attainment.

### 3.3. Childhood Fish Consumption, by Fish Type

Most fish consumers reported typically consuming canned tuna (54%) or white fish (e.g., fish sticks, cod, and haddock, 33%); high-mercury fish (e.g., bluefish, fresh tuna, and swordfish, 6%) and other fish (e.g., flatfish, striped bass, and salmon, 7%) were also reported. Participants who typically ate high-mercury fish were more likely to have educated mothers and fathers with white-collar employment than non-fish consumers or typical consumers of low-mercury fish or canned tuna (Table S2). Participants who typically ate high-mercury fish also tended to eat fish slightly more frequently, with 61% of high-mercury fish consumers eating fish about once a week or more, compared to 50% of low-mercury fish and canned tuna eaters. When we modeled the type of fish typically consumed as the exposure, we observed an increased odds of ADD/ADHD for consumers of each fish type relative to non-fish consumers (for low-mercury fish, OR: 3.3, 95% CI: 1.0–11; for canned tuna, OR: 3.1, 95% CI: 0.9–10; for high-mercury fish, OR: 4.4, 95% CI: 1.1–18) (Table S3). The effect estimate for high-mercury fish was not significantly greater than that for low-mercury fish. Typical consumers of low-mercury fish (OR: 2.1, 95% CI: 1.0–4.3) and canned tuna (OR: 1.7, 95% CI: 0.8–3.5), but not high-mercury fish (OR: 0.9; 95% CI: 0.3–3.1), also had an elevated odds of repeating a grade relative to non-fish consumers. Small sample sizes did not permit us to jointly consider type and frequency of fish consumption.

### 3.4. Frequency of High-Mercury Fish Consumption

**Figure 1:**
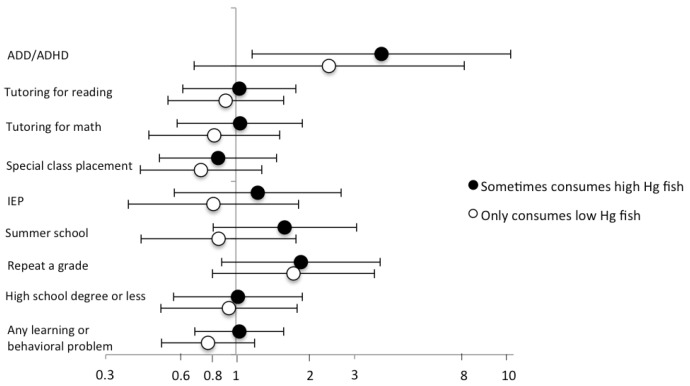
Multivariable ^a^ GEE odds ratios for associations of childhood (ages 7–12 years) fish consumption with learning and behavioral problems according to frequency of high-mercury fish ^b^ consumption. Participants who reported no childhood fish consumption (N = 122) were considered the reference. ^a^ Models adjusted for maternal age at birth (≤21, 22–25, 26–29, ≥30 years), maternal education at time of birth (high school diploma or less, some college, 4-year college grad or higher), and participant race (white, other), sex, year of birth (1969–1974, 1975–1980, 1981–1983), and combined gestational age/birthweight (preterm or <2500 g, term and ≥2500 g); ^b^ Including fresh tuna, swordfish, bluefish, and freshwater bass. Abbreviations: ADD: attention deficit disorder; ADHD, attention deficit hyperactivity disorder; CI, confidence interval; GEE, generalized estimating equation; OR, odds ratio.

Although only 6% of participants typically ate high-mercury fish, another 50% of fish eaters reported occasional high-mercury fish consumption. More so than for other exposure metrics, frequency of high-mercury fish consumption was positively associated with markers of socioeconomic status, particularly maternal education and paternal employment (Table 4). When we compared participants who sometimes ate high-mercury fish and those who only ate low-mercury fish to non-fish consumers, high-mercury fish eaters had the highest odds of every outcome (Figure 1). However, with the exception of ADD/ADHD, associations were not statistically significant. We were unable to obtain stable effect estimates for occasional and typical high-mercury fish consumption separately due to small sample sizes.

### 3.5. Sensitivity Analyses

To evaluate potential diagnostic bias (i.e., greater likelihood of diagnosis or access to services among higher SES participants), we stratified by maternal education. Our findings did not meaningfully differ between groups defined by maternal education (data not shown).

## 4. Discussion

In this retrospective study of self-reported childhood fish consumption and learning and behavioral problems, we found that fish consumption was not associated with learning and behavioral problems, with the exception of a positive association with ADD/ADHD. The ADD/ADHD association was strongest for typical consumers of high-mercury fish, but, unexpectedly, also observed for those who typically chose low-mercury fish or canned tuna. The low-mercury fish varieties reported by our participants were typically low in n-3 PUFA [20], and therefore may not be expected to yield any particular cognitive benefit; however, it is unclear why consumption of relatively low-mercury fish would adversely impact learning abilities and behavioral disorders. Although statistically significant, our ADD/ADHD findings were characterized by wide confidence intervals indicating a lack of statistical precision, and should be repeated in a larger population with a more stable reference group.

### 4.1. Previous Studies

Despite study populations differing in fish consumption and age at exposure among other factors, previous studies have generally indicated a beneficial effect of postnatal fish consumption on learning and behavioral outcomes. In a prospective study of Swedish adolescents, frequency of fish consumption at age 15 years was positively associated with cognitive performance in 3972 18-year old boys [9] and academic attainment in 9448 16-year boys and girls [13]. Infants enrolled in a British birth cohort (N = 7421) who consumed fish at least once weekly had higher language comprehension/ language and social activity scores than those who did not consume fish, with similar findings observed when oily and white fish intake were modeled separately [10]. Type of fish did appear to matter in a cross-sectional study of 75 Spanish preschoolers in which more frequent consumption of oily and canned fish was associated with a better general cognitive score (a composite measure of quantitative, memory, verbal, perceptual-performance, and motor domains) while the opposite was observed for white and fried fish [12]. Other studies have modeled a single nutrient or contaminant of fish as the exposure, an approach that, while neglecting the possibly opposing effects of other constituents, captures some of the variability observed across fish species. In a cross-sectional analysis of 700 Dutch 12 to 18 year-olds, the authors estimated n-3 PUFA intake from a report of fish consumption that included type of fish [11]. n-3 PUFA intake was positively associated with vocabulary scores and higher academic grades until the recommended n-3 PUFA intake was reached, after which inverse associations were observed. Several studies have investigated the association between postnatal blood mercury and ADHD diagnosis or symptomology [27,28,29]. No associated between blood mercury and ADHD was reported in a cross-sectional study of 83 Romanian children [29] or a case-control study of 129 U.S. children living near a former lead refinery [28]; however, reported blood mercury concentrations were low relative to populations with regular fish consumption and may have been inadequate to detect an association with ADHD. Higher blood mercury concentrations were observed in a cross-sectional study of 1778 South Korean children [27]; however, this study also failed to detect an association between blood mercury and ADHD symptoms [27]. Although fish consumption in this study was not reported, fish is a popular food in South Korea, and the authors suggest that a mercury-ADHD association could have been obscured by high n-3 PUFA consumption.

### 4.2. Strengths and Limitations

Participants were 23–41 years of age when they recalled their childhood fish consumption and childhood and adolescent learning and behavioral problems, making some exposure and outcome misclassification likely. However, we would not expect participants who also reported childhood learning or behavioral problems to remember their childhood fish consumption differently than those without such problems or vice versa (i.e., recall bias). Prenatal mercury exposure has been reported to be associated with memory deficits [30], raising the possibility that participants who consumed high-mercury fish as children may recall their childhood fish consumption or learning and behavioral problems relatively less accurately. However, this concern is somewhat mitigated by the high degree of agreement we found between participants’ reports of learning and behavioral problems with those reported by their mothers. Moreover, fishing is a major aspect of Cape Cod industry and culture, which we suspect would improve our participants’ knowledge of the varieties of fish they ate. We categorized participants based on their consumption of high-mercury fish because we considered this contaminant most relevant with respect to public health practice, specifically current FDA/EPA fish consumption recommendations for pregnant women, nursing mothers, and young children [21]. The limitation of this approach is that it fails to consider other potentially important nutrients, particularly n-3 PUFA, and contaminants (e.g., polychlorinated biphenyls) that vary between species of fish. Finally, we were limited by the absence of human mercury biomarkers, which would have been helpful in supporting our exposure cutpoints and could have facilitated comparisons to other cohorts.

We observed that higher SES children consumed high-mercury fish more frequently than low SES children, possibly because higher SES families had more leisure time to pursue sport fishing or could afford more expensive varieties of fish (e.g., swordfish). We therefore considered the possibility that some of our results may be effected by a diagnostic or ascertainment bias, in which children with more frequent high-mercury fish consumption may have had greater access to services (e.g., tutoring) creating an upwards bias. However, this hypothesis was not supported by our findings, which did not vary by maternal education, a marker of SES that appeared strongly associated with high-mercury fish consumption in this sample. We similarly expected that socioeconomically disadvantaged children may have been more likely to be diagnosed as ADD/ADHD due to teacher or clinician bias [31], which could lead to a spurious association between low-mercury fish consumption and ADD/ADHD. We observed similar findings for ADD/ADHD symptoms (i.e., difficulty sitting still or paying attention compared to peers) and diagnosis, which suggests that these results were not in large part explained by diagnostic bias.

We adjusted for many predictors of learning and behavioral problems, but residual or unmeasured confounding is still possible. We did not measure mercury biomarkers or assess other aspects of dietary intake, precluding our ability to adjust for total energy, nutrient intake, or other foods typically eaten in combination with fish (e.g., French fries). Further, we did not collect information on maternal fish consumption during the participant’s gestation or the participant’s fish consumption during early childhood, which may also be a critical period of brain development [7].

Consistent with the importance of fishing in Cape Cod, fish intake among cohort participants was more frequent than otherwise seen in the US. Approximately 90% of our participants reported some childhood fish consumption compared to 51% of 6 to 11 year old National Health and Nutrition Examination Survey participants reporting fish consumption within the last month between 1999 and 2006 [32]. With the exception of salmon, which was not popular in our cohort, Cape Cod children tended to eat the same varieties of fish (e.g., canned tuna and breaded fish) as other U.S. children [32].

We considered a participant to have a “learning or behavioral problem” if they reported one or more of our 8 outcomes of interest. The high number of participants reporting a learning or behavioral problem (N = 451, 43%) is likely a function of the number of included outcomes as well as the extended time frame (any time during grade school) over which the problem could have occurred. In addition, temporal changes have been noted in ADD/ADHD diagnostic practices [33,34], and study participants tended to report a later age at diagnosis than would be expected in contemporary children. However, the number of adults in our cohort who reported being diagnosed with ADD/ADHD (~7%) was consistent with an estimated prevalence of 7%–12% in the U.S. [35,36], and our findings for ADD/ADHD were similar to those for the outcome of “difficulty paying attention or sitting still compared to peers,” which does not rely on diagnosis. The percentage of participants who reported obtaining a high school diploma or less (12%) was lower than Census estimates of 33% (for those currently 25–34 years of age), but in keeping with higher educational attainment for non-Hispanic whites, U.S. nationals, and Massachusetts residents [37]. We therefore expect that our findings are generalizable to contemporary children with similar levels of fish consumption.

## 5. Conclusions

The FDA/EPA takes species-specific variability in mercury into consideration by recommending that pregnant women, nursing mothers, and young children limit consumption or completely avoid high-mercury fish. Despite this, epidemiologic research on the health effects of fish consumption is usually limited to overall, rather than species-specific, fish consumption. In this unique Cape Cod population with regular and diverse fish consumption, we found no association between childhood consumption of fish, including high-mercury varieties, and learning or behavioral problems, with the exception of a positive association with ADD/ADHD. Although participants in our study tended to eat more fish than other U.S. children, only 11% of our population met the FDA/EPA recommended maximum consumption level of two to three times a week [21], and few would have consumed the quantity and quality of fish recommended for maximum n-3 PUFA benefit [20]. Thus, while our findings generally support the safety of the observed level of fish consumption, the absence of a beneficial effect may be attributed to insufficient fish intake and/or choice of relatively low n-3 PUFA fish. We considered fish consumption between 7–12 years only; future studies should additionally consider maternal diet during pregnancy and early childhood diet.

## Figures and Tables

**Table 1:** Eligibility of study participants, by childhood (ages 7–12 years) fish consumption (N (%)).

Exclusion Criteria	Fish Consumption (N = 1526)	No Fish Consumption (N = 163)	Total (N = 1689)
Not included in previous study ^a^	294 (19.3) ^a^	24 (14.7)	318 (18.8)
Missing data on childhood fish consumption	58 (3.8) ^b^	0 (0.0)	73 (4.3)
Multiple birth	28 (1.8)	7 (4.3)	35 (2.1)
Prenatal exposure to ≥7 alcoholic drinks/week	19 (1.3)	3 (1.8)	22 (1.3)
Prenatal exposure to known teratogen	7 (0.5)	1 (0.6)	8 (0.5)
History of deafness or blindness	13 (0.9)	3 (1.8)	16 (0.9)
Mental retardation	4 (0.3)	0 (0.0)	4 (0.2)
Lead poisoning	6 (0.4)	0 (0.0)	6 (0.4)
Missing data on key covariates	25 (1.6)	3 (1.8)	28 (1.7)
Total eligible ^c^	1057 (69.3)	122 (74.9)	1179 (69.8)

^a^ Maternal questionnaire administered in previous study used to obtain data on prenatal exposures; ^b^ 15 additional participants were missing all data on childhood fish consumption and therefore unable to be classified as fish consumers or non-consumers; ^c^ Percentages do not sum to 100 due to rounding.

**Table 2:** Characteristics of 1179 participants, by overall childhood (ages 7–12 years) fish consumption (N (%)).

Characteristic	Childhood Fish Consumption (Meals)				
	None (N = 122)	Once a Month or Less (N = 153)	A Couple of Times a Month (N = 356)	About Once a Week (N = 423)	Several Times a Week (N = 125)
Year of birth					
1969–1974	21 (17.2)	29 (19.0)	81 (22.8)	122 (28.8)	44 (35.2)
1975–1980	72 (59.0)	78 (51.0)	184 (51.7)	203 (48.0)	59 (47.2)
1981–1983	29 (23.8)	46 (30.1)	91 (25.6)	98 (23.2)	22 (17.6)
Current age (years), mean ± SD	29.1 ± 3.5	28.9 ± 3.8	29.6 ± 4.0	30.0 ± 3.9	30.7 ± 3.9
Male	52 (42.6)	52 (34.0)	145 (40.7)	168 (39.7)	54 (43.2)
White race	122 (100.0)	153 (100.0)	349 (98.0)	416 (98.4)	123 (98.4)
Birthweight (grams), mean ± SD ^a,b^	3480 ± 537	3507 ± 500	3458 ± 471	3460 ± 508	3451 ± 494
Preterm (<37 weeks gestation)	8 (6.6)	4 (2.6)	18 (5.1)	20 (4.7)	4 (3.2)
Participant was breastfed	70 (57.4)	94 (62.3)	222 (63.6)	273 (66.4)	76 (62.3)
Current level of education					
High school graduate or less	17 (13.9)	27 (17.7)	38 (10.7)	42 (9.9)	20 (16.0)
Some college	37 (30.3)	32 (20.9)	96 (27.0)	88 (20.8)	33 (26.4)
≥4 years of college	68 (55.7)	94 (61.4)	221 (62.3)	293 (69.3)	72 (57.6)
Mother’s age at participant’s birth (years), mean ± SD	27.4 ± 4.3	26.6 ± 4.3	27.2 ± 4.7	27.2 ± 4.4	26.3 ± 4.8
Father’s age at participant’s birth (years), mean ± SD	30.1 ± 6.2	28.2 ± 4.5	29.7 ± 5.8	29.9 ± 5.5	28.6 ± 5.6
Mother’s education level at participant’s birth					
High school graduate or less	49 (40.2)	56 (36.6)	131 (36.8)	123 (29.1)	44 (35.2)
Some college	41 (33.6)	48 (31.4)	107 (30.1)	137 (32.4)	38 (30.4)
≥4 years of college	32 (26.2)	49 (32.0)	118 (33.2)	163 (38.5)	43 (34.4)
Father’s occupation at participant’s birth					
White collar	59 (49.2)	71 (46.4)	178 (50.6)	237 (57.0)	58 (46.8)
Blue collar	39 (32.5)	49 (32.0)	103 (29.3)	126 (30.3)	44 (35.5)
Other	22 (18.3)	33 (21.6)	71 (20.2)	53 (12.7)	22 (17.7)
Mother received prenatal care during participant’s gestation	121 (100.0)	150 (99.3)	352 (99.4)	418 (99.8)	125 (100.0)
Maternal smoking during pregnancy					
None	83 (68.6)	118 (77.1)	260 (73.9)	310 (74.0)	86 (69.4)
Smoked ≤ 10 cigarettes a day	12 (9.9)	15 (9.8)	48 (13.6)	47 (11.2)	15 (12.1)
Smoked ≥ 11 cigarettes a day	26 (21.5)	20 (13.1)	44 (12.5)	62 (14.8)	23 (18.6)
Maternal alcohol consumption during pregnancy					
None	66 (54.6)	93 (61.6)	198 (56.4)	214 (51.0)	69 (55.7)
1–3 drinks/month	36 (29.8)	42 (27.8)	105 (29.9)	130 (31.0)	36 (29.0)
≥1 drink/week	19 (15.7)	16 (10.6)	48 (13.7)	76 (18.1)	19 (15.3)
Family history of ADD/ADHD	25 (21.0)	19 (12.9)	56 (16.1)	52 (12.7)	26 (21.7)
Family history of learning disabilities	22 (19.0)	21 (14.0)	77 (22.3)	76 (19.0)	31 (25.2)
Sometimes consumed high-mercury fish	0 (0.0)	46 (30.1)	172 (48.3)	279 (66.0)	99 (79.2)
Both pre- and postnatal perchloroethylene (PCE) exposure	70 (66.7)	85 (65.9)	158 (55.1)	221 (62.6)	61 (61.0)

^a^ Information on birthweight, gestational age, parental age, maternal education, and prenatal care were obtained from birth records or questionnaires completed by participants’ mothers; ^b^ Missing: highest education level of participant (N = 1), paternal occupation (N = 14), prenatal care (N = 9), maternal smoking (N = 10), family history of ADD/ADHD (N = 36), family history of learning disabilities (N = 43), PCE exposure (N = 205). Abbreviations: ADD, attention deficit disorder; ADHD, attention deficit hyperactivity disorder; SD, standard deviation.

**Table 3:** Overall fish consumption during childhood (ages 7–12 years) and odds of learning and behavioral problems (N = 1179).

OR (95% CI)	Childhood Fish Consumption (Meals)					
	None (N = 122)	Once a Month or Less (N = 153)	A Couple Times a Month (N = 356)	About Once a Week (N = 423)	Several Times a Week (N = 125)	*p*-Trend ^a^
ADD/ADHD						
Events/N	3/117	12/153	21/353	29/421	13/123	
Model 1: Crude logistic	1.0 (Reference)	3.2 (0.9–12)	2.4 (0.7–8.2)	2.8 (0.8–9.4)	4.5 (1.2–16)	0.04
Model 2: Unadjusted GEE	1.0 (Reference)	3.2 (0.9–12)	2.4 (0.7–8.1)	2.8 (0.8–9.3)	4.5 (1.2–16)	0.04
Model 3: Adjusted GEE ^b^	1.0 (Reference)	3.8 (1.1–13)	2.6 (0.8–8.8)	3.0 (0.9–9.7)	5.2 (1.5–18)	0.02
Tutoring for reading						
Events/N	20/122	18/152	56/355	62/417	24/124	
Model 1: Crude logistic	1.0 (Reference)	0.7 (0.3–1.4)	1.0 (0.5–1.7)	0.9 (0.5–1.5)	1.2 (0.6–2.4)	0.23
Model 2: Unadjusted GEE	1.0 (Reference)	0.7 (0.4–1.3)	1.0 (0.5–1.7)	0.9 (0.5–1.5)	1.2 (0.6–2.3)	0.26
Model 3: Adjusted GEE ^b^	1.0 (Reference)	0.8 (0.4–1.5)	1.0 (0.6–1.7)	1.0 (0.6–1.6)	1.3 (0.7–2.6)	0.19
Tutoring for math						
Events/N	18/114	18/150	53/342	51/404	24/124	
Model 1: Crude logistic	1.0 (Reference)	0.7 (0.4–1.5)	1.0 (0.5–1.8)	0.8 (0.4–1.4)	1.3 (0.7–2.5)	0.23
Model 2: Unadjusted GEE	1.0 (Reference)	0.8 (0.4–1.6)	1.0 (0.5–1.8)	0.8 (0.4–1.4)	1.3 (0.6–2.5)	0.33
Model 3: Adjusted GEE ^b^	1.0 (Reference)	0.8 (0.4–1.6)	1.0 (0.5–1.9)	0.8 (0.4–1.5)	1.3 (0.7–2.7)	0.26
Special class placement ^c^						
Events/N	20/122	23/152	45/353	43/419	22/125	
Model 1: Crude logistic	1.0 (Reference)	0.9 (0.5–1.7)	0.7 (0.4–1.3)	0.6 (0.3–1.0)	1.1 (0.6–2.1)	0.56
Model 2: Unadjusted GEE	1.0 (Reference)	0.9 (0.5–1.8)	0.7 (0.4–1.3)	0.6 (0.3–1.0)	1.1 (0.6–2.1)	0.59
Model 3: Adjusted GEE ^b^	1.0 (Reference)	1.0 (0.5–2.0)	0.8 (0.4–1.3)	0.6 (0.4–1.1)	1.1 (0.6–2.2)	0.52
Individualized Education Plan						
Events/N	9/121	11/152	25/353	28/417	11/125	
Model 1: Crude logistic	1.0 (Reference)	1.0 (0.4–2.4)	0.9 (0.4–2.1)	0.9 (0.4–2.0)	1.2 (0.5–3.0)	0.59
Model 2: Unadjusted GEE	1.0 (Reference)	1.0 (0.4–2.6)	1.0 (0.4–2.2)	0.9 (0.4–2.0)	1.2 (0.5–3.1)	0.64
Model 3: Adjusted GEE ^b^	1.0 (Reference)	1.1 (0.4–2.8)	1.0 (0.4–2.2)	1.0 (0.4–2.2)	1.3 (0.5–3.4)	0.54
Attend summer school						
Events/N	11/121	16/153	37/356	44/418	15/124	
Model 1: Crude logistic	1.0 (Reference)	1.2 (0.5–2.6)	1.2 (0.6–2.4)	1.2 (0.6–2.4)	1.4 (0.6–3.1)	0.49
Model 2: Unadjusted GEE	1.0 (Reference)	1.1 (0.6–2.3)	1.2 (0.6–2.3)	1.3 (0.5–2.9)	1.1 (0.6–2.3)	0.65
Model 3: Adjusted GEE ^b^	1.0 (Reference)	1.3 (0.6–3.0)	1.2 (0.6–2.4)	1.2 (0.6–2.5)	1.3 (0.5–3.0)	0.72
Repeat a grade						
Events/N	9/120	12/152	41/354	55/417	16/125	
Model 1: Crude logistic	1.0 (Reference)	1.1 (0.4–2.6)	1.6 (0.8–3.4)	1.9 (0.9–3.9)	1.8 (0.8–4.3)	0.18
Model 2: Unadjusted GEE	1.0 (Reference)	1.6 (0.8–3.5)	1.9 (0.9–3.9)	1.8 (0.8–4.3)	1.6 (0.8–3.5)	0.16
Model 3: Adjusted GEE ^b^	1.0 (Reference)	1.2 (0.5–2.9)	1.7 (0.8–3.6)	2.1 (1.0–4.4)	1.9 (0.8–4.5)	0.18
High school degree or less						
Events/N	17/122	27/153	38/355	42/423	20/125	
Model 1: Crude logistic	1.0 (Reference)	1.3 (0.7–2.6)	0.7 (0.4–1.4)	0.7 (0.4–1.2)	1.2 (0.6–2.4)	0.67
Model 2: Unadjusted GEE	1.0 (Reference)	1.2 (0.6–2.5)	0.7 (0.4–1.4)	0.7 (0.4–1.2)	1.2 (0.6–2.3)	0.72
Model 3: Adjusted GEE ^b^	1.0 (Reference)	1.4 (0.7–2.8)	0.8 (0.4–1.6)	0.9 (0.5–1.6)	1.4 (0.6–3.0)	0.39

^a^ Modeled as a continuous variable; ^b^ Models adjusted for maternal age at birth (≤21, 22–25, 26–29, ≥30 years), maternal education at time of birth (high school diploma or less, some college, 4-year college grad or higher), and participant race (white, other), sex, year of birth (1969–1974, 1975–1980, 1981–1983), and combined gestational age/birthweight (preterm or <2500 g, term and ≥2500 g); ^c^ Assigned to a special class because of academic or behavioral problems. Abbreviations: ADD: attention deficit disorder; ADHD, attention deficit hyperactivity disorder; CI, confidence interval; GEE, generalized estimating equation; OR, odds ratio.

**Table 4:** Characteristics of 1179 participants, by frequency of high-mercury fish consumption during childhood (ages 7–12 years) (N (%)).

	No Fish Consumption (N = 122)	Eat Fish, Never High-Mercury Varieties (N = 460)	Eat Fish, Occasionally High-Mercury Varieties (N = 535)	Eat Fish, Typically High-Mercury Varieties (N = 62)
Year of birth				
1969–1974	21 (17.2)	119 (25.9)	141 (26.4)	16 (25.8)
1975–1980	72 (59.0)	243 (52.8)	255 (47.7)	26 (41.9)
1981–1983	29 (23.8)	98 (21.3)	139 (26.0)	20 (32.3)
Current age (years), mean ± SD	29.1 ± 3.5	29.9 ± 3.8	29.7 ± 4.0	29.2 ± 3.9
Male	52 (42.6)	145 (31.5)	243 (45.4)	31 (50.0)
White race	122 (100)	453 (98.5)	526 (98.3)	62 (100)
Birthweight (grams), mean ± SD ^a,b^	3480 ± 537	3446 ± 512	3479 ± 489	3482 ± 458
Preterm (<37 weeks gestation)	8 (6.6)	20 (4.4)	22 (4.1)	4 (6.5)
Participant was breastfed	70 (57.4)	283 (63.0)	340 (65.0)	42 (68.9)
Current level of education				
High school graduate or less	17 (13.9)	56 (12.2)	64 (12.0)	7 (11.3)
Some college	37 (30.3)	127 (27.7)	109 (20.4)	13 (21.0)
≥4 years of college	68 (55.7)	276 (60.1)	362 (67.7)	42 (67.7)
Mother’s age at participant’s birth (years), mean ± SD	27.4 ± 4.3	26.7 ± 4.5	27.3 ± 4.5	27.4 ± 4.4
Father’s age at participant’s birth (years), mean ± SD	30.1 ± 6.2	28.9 ± 5.3	29.8 ± 5.7	29.8 ± 5.4
Mother’s education level at participant’s birth				
High school graduate or less	49 (40.2)	179 (38.9)	160 (29.9)	15 (24.2)
Some college	41 (33.6)	145 (31.5)	167 (31.2)	18 (29.0)
≥4 years of college	32 (26.2)	136 (29.6)	208 (38.9)	29 (46.8)
Father’s occupation at participant’s birth				
White collar	59 (49.2)	207 (45.6)	301 (56.9)	36 (58.1)
Blue collar	39 (32.5)	153 (33.7)	153 (28.9)	16 (25.8)
Other	22 (18.3)	94 (20.7)	75 (14.2)	10 (16.1)
Mother received prenatal care during participant’s gestation	121 (100)	454 (99.6)	530 (99.6)	61 (100)
Maternal smoking during pregnancy				
None	83 (68.6)	338 (74.1)	393 (74.0)	43 (70.5)
Smoked ≤ 10 cigarettes a day	12 (9.9)	58 (12.7)	58 (10.9)	9 (14.8)
Smoked ≥ 11 cigarettes a day	26 (21.5)	60 (13.2)	80 (15.1)	9 (14.8)
Maternal alcohol consumption during pregnancy				
None	66 (54.6)	263 (57.8)	278 (52.5)	33 (54.1)
1–3 drinks/month	36 (29.8)	137 (30.1)	155 (29.3)	21 (34.4)
≥1 drink/week	19 (15.7)	55 (12.1)	97 (18.3)	7 (11.5)
Family history of ADD/ADHD	25 (21.0)	58 (12.9)	85 (16.5)	10 (17.0)
Family history of learning disabilities	22 (19.0)	83 (18.5)	112 (21.8)	10 (17.0)

^a^ Information on birthweight, gestational age, parental age, maternal education, and prenatal care were obtained from birth records or questionnaires completed by participants’ mothers; ^b^ Missing: highest education level of participant (N = 1), paternal occupation (N = 14), prenatal care (N = 9), maternal smoking (N = 10), family history of ADD/ADHD (N = 36), family history of learning disabilities (N = 43); Abbreviations: ADD, attention deficit disorder; ADHD, attention deficit hyperactivity disorder; SD, standard deviation.

## Supplementary Materials

The following are available online at www.mdpi.com/1660-4601/13/11/1069/s1, Table S1: Varieties of fish typically consumed by participants in the Cape Cod Health Study reporting childhood (ages 7–12 years) fish consumption (N = 1057), according to mercury levels previously published by the FDA, Table S2: Characteristics of 1179 participants, by typical variety of fish consumed during childhood (N (%)), Table S3: Variety of fish typically consumed during childhood (ages 7–12 years) and odds of learning and behavioral problems (N = 1179).
