# Supplementary material for: Childhood Fish Consumption and Learning and Behavioral Disorders
Source: Int J Environ Res Public Health. 2016 Nov 2;13(11):1069. doi: 10.3390/ijerph13111069 (PMC5129279; doi:10.3390/ijerph13111069)
Supplement: Supplementary file 1 [file ijerph-13-01069-s001.pdf]

Supplementary Materials: Childhood Fish Consumption and Learning and Behavioral Disorders

Jenny L. Carwile, Lindsey J. Butler, Patricia A. Janulewicz, Michael R. Winter and Ann Aschengrau

Table S1. Varieties of fish typically consumed by participants in the Cape Cod Health Study reporting childhood (ages 7–12 years) fish consumption (N = 1057), according to mercury levels previously published by the FDA [a].

Variety of Fish	N (%) [b]	Mercury, Mean (μg/g)	2004 FDA/EPA Consumption Advice [b]
Low-mercury			Up to 2 meals/week
Fish sticks, frozen	59 (5.6)	0.003 [c]	
Salmon, fresh/frozen	13 (1.2)	0.02 [d]	
Pollock	1 (0.1)	0.04 [d]	
Haddock	62 (5.9)	0.07 [d]	
Bass, striped	17 (1.6)	0.07 [d]	
Flatfish	45 (3.7)	0.08 [d]	
Cod/ scrod	224 (21.2)	0.09 [d]	
Mullet	1 (0.1)	0.15 [d]	
Halibut	4 (0.4)	0.22 [d]	
Canned tuna	569 (53.8)	Chunk light: 0.12 [d,e]<br>Albacore: 0.35 [d,e]	Up to 2 meals/week<br>Up to 1 meal/week
High-mercury			
Bass, freshwater	1 (0.1)	0.32 [d]	
Bluefish	30 (2.8)	0.35 [d]	
Tuna, fresh, all species	2 (0.2)	0.39 [d]	
Swordfish	29 (2.7)	1.00 [d]	Avoid

[a] Mercury levels vary within species according to factors such as size and age of the fish and geographic location; [b] Percentages do not sum to 100 due to rounding; [c] Fish: what pregnant women and parents should know. Draft updated advice by the FDA and EPA/June 2014 [1]; [d] U.S. Food and Drug Administration. 2014 [2]; [e] Variety of canned tuna was not reported.

Table S2. Characteristics of 1179 participants, by typical variety of fish consumed during childhood (N (%)) [a].

Characteristic	Fish Typically Consumed during Childhood			
	None (N = 122)	Low-Mercury Fish (N = 425)	Canned Tuna (N = 570)	High-Mercury Fish (N = 62)
Year of birth				
1969–1974	21 (17.2)	116 (29.3)	144 (25.3)	16 (25.8)
1975–1980	72 (59.0)	213 (50.1)	285 (50.0)	26 (41.9)
1981–1983	29 (23.8)	96 (22.6)	141 (24.7)	20 (32.3)
Current age (years), mean ± SD	29.1 ± 3.5	29.9 ± 3.9	29.7 ± 3.9	29.2 ± 3.9
Male	52 (42.6)	179 (42.1)	209 (36.7)	31 (50.0)
White race	122 (100.0)	418 (98.4)	561 (98.4)	62 (100.0)
Birthweight (grams), mean ± SD	3480 ± 537	3470 ± 506	3459 ± 496	3482 ± 458
Preterm (<37 weeks gestation)	8 (6.6)	16 (3.8)	26 (4.6)	4 (6.5)
Participant was breastfed	70 (57.4)	268 (64.7)	355 (63.6)	42 (68.9)
Current level of education				
High school graduate or less	17 (13.9)	52 (12.2)	68 (12.0)	7 (11.3)
Some college	37 (30.3)	105 (24.7)	131 (23.0)	13 (21.0)
≥4 years of college	68 (55.7)	268 (63.1)	370 (65.0)	42 (67.7)

Table S2. *Cont.*

	Fish Typically Consumed during Childhood			
	**None (N = 122)**	**Low-Mercury Fish (N = 425)**	**Canned Tuna (N = 570)**	**High-Mercury Fish (N = 62)**
Mother's age at participant's birth (years), mean ± SD	27.4 ± 4.3	26.9 ± 4.5	27.1 ± 4.5	27.4 ± 4.4
Father's age at participant's birth (years), mean ± SD	30.1 ± 6.2	29.5 ± 5.7	29.3 ± 5.4	29.8 ± 5.4
Mother's education level at participant's birth				
High school graduate or less	49 (40.2)	140 (32.9)	199 (34.9)	15 (24.2)
Some college	41 (33.6)	140 (32.9)	172 (30.2)	18 (29.0)
≥4 years of college	32 (26.2)	145 (34.1)	199 (34.9)	29 (46.8)
Father's occupation at participant's birth				
White collar	59 (49.2)	212 (50.6)	296 (52.5)	36 (58.1)
Blue collar	39 (32.5)	139 (33.2)	167 (29.6)	16 (25.8)
Other	22 (18.3)	68 (16.2)	101 (17.9)	10 (16.3)
Mother received prenatal care during participant's gestation	121 (100.0)	421 (99.5)	563 (99.7)	61 (100.0)
Maternal smoking during pregnancy				
None	83 (68.6)	309 (73.2)	422 (74.7)	43 (70.5)
Smoked ≤ 10 cigarettes a day	12 (9.9)	50 (11.9)	66 (11.7)	9 (14.8)
Smoked ≥ 11 cigarettes a day	26 (21.5)	63 (14.9)	77 (13.6)	9 (14.8)
Maternal alcohol consumption during pregnancy				
None	66 (54.6)	225 (53.3)	316 (56.1)	33 (54.1)
1–3 drinks/month	36 (29.8)	134 (31.8)	158 (28.1)	21 (34.4)
≥1 drink/week	19 (15.7)	63 (14.9)	89 (15.8)	7 (11.5)
Family history of ADD/ADHD	25 (21.0)	67 (16.2)	76 (13.8)	10 (17.0)
Family history of learning disabilities	22 (19.0)	99 (24.2)	96 (17.4)	10 (17.0)

[a] Missing: highest education level of participant (N = 1), paternal occupation (N = 13), prenatal care (N = 8), maternal smoking (N = 10), family history of ADD/ADHD (N = 36), family history of learning disabilities (N = 42). Abbreviations: ADD, attention deficit disorder; ADHD, attention deficit hyperactivity disorder; SD, standard deviation.

Table S3. Variety of fish typically consumed during childhood (ages 7–12 years) and odds of learning and behavioral problems (N = 1179).

**OR and (95% CI)**	Fish Typically Consumed during Childhood [a]			
	**None (N = 122)**	**Low-Mercury Fish (N = 425)**	**Canned Tuna (N = 570)**	**High-Mercury Fish (N = 62)**
ADD/ADHD				
Events/N	3/117	31/422	37/566	7/62
Model 1: Crude logistic	1.0 (Reference)	3.0 (0.9–10)	2.7 (0.8–8.8)	4.8 (1.2–19)
Model 2: Unadjusted GEE	1.0 (Reference)	3.1 (0.9–11)	2.7 (0.8–9.3)	5.0 (1.2–21)
Model 3: Adjusted GEE [a]	1.0 (Reference)	3.3 (1.0–11)	3.1 (0.9–10)	4.4 (1.1–18)
Tutoring for reading				
Events/N	20/122	69/422	82/565	9/61
Model 1: Crude logistic	1.0 (Reference)	1.0 (0.6–1.7)	0.9 (0.5–1.5)	0.9 (0.4–2.1)
Model 2: Unadjusted GEE	1.0 (Reference)	1.0 (0.6–1.7)	0.9 (0.5–1.4)	0.9 (0.4–2.1)
Model 3: Adjusted GEE [a]	1.0 (Reference)	1.0 (0.6–1.8)	0.9 (0.6–1.6)	1.0 (0.4–2.3)
Tutoring for math				
Events/N	18/114	63/410	77/548	6/62
Model 1: Crude logistic	1.0 (Reference)	1.0 (0.5–1.7)	0.9 (0.5–1.5)	0.6 (0.2–1.5)
Model 2: Unadjusted GEE	1.0 (Reference)	1.0 (0.5–1.8)	0.9 (0.5–1.6)	0.6 (0.2–1.6)
Model 3: Adjusted GEE [a]	1.0 (Reference)	1.0 (0.6–1.8)	0.9 (0.5–1.7)	0.6 (0.2–1.7)
Special class placement [b]				
Events/N	20/122	53/420	73/567	7/62
Model 1: Crude logistic	1.0 (Reference)	0.7 (0.4–1.3)	0.8 (0.4–1.3)	0.6 (0.3–1.6)
Model 2: Unadjusted GEE	1.0 (Reference)	0.7 (0.4–1.3)	0.8 (0.4–1.3)	0.7 (0.3–1.7)
Model 3: Adjusted GEE [a]	1.0 (Reference)	0.8 (0.4–1.4)	0.8 (0.5–1.4)	0.7 (0.3–1.9)

Table S3. *Cont.*

	Fish Typically Consumed during Childhood [a]			
	**None (N = 122)**	**Low-Mercury Fish (N = 425)**	**Canned Tuna (N = 570)**	**High-Mercury Fish (N = 62)**
Individualized Education Plan				
Events/N	9/121	33/421	38/564	4/62
Model 1: Crude logistic	1.0 (Reference)	1.1 (0.5–2.3)	0.9 (0.4–1.9)	0.9 (0.3–2.9)
Model 2: Unadjusted GEE	1.0 (Reference)	1.1 (0.5–2.4)	0.9 (0.4–2.0)	0.9 (0.3–3.2)
Model 3: Adjusted GEE [a]	1.0 (Reference)	1.1 (0.5–2.5)	1.0 (0.5–2.1)	1.0 (0.3–3.3)
Attend summer school				
Events/N	11/121	47/421	58/568	7/62
Model 1: Crude logistic	1.0 (Reference)	1.3 (0.6–2.5)	1.1 (0.6–2.2)	1.3 (0.5–3.5)
Model 2: Unadjusted GEE	1.0 (Reference)	1.2 (0.6–2.4)	1.1 (0.6–2.2)	1.2 (0.5–3.1)
Model 3: Adjusted GEE [a]	1.0 (Reference)	1.3 (0.6–2.5)	1.2 (0.6–2.4)	1.3 (0.5–3.4)
Repeat a grade				
Events/N	9/120	56/421	63/565	4/62
Model 1: Crude logistic	1.0 (Reference)	1.9 (0.9–4.0)	1.5 (0.7–3.2)	0.9 (0.3–2.9)
Model 2: Unadjusted GEE	1.0 (Reference)	1.9 (0.9–4.0)	1.6 (0.7–3.2)	0.9 (0.2–2.9)
Model 3: Adjusted GEE [a]	1.0 (Reference)	2.1 (1.0–4.3)	1.7 (0.8–3.5)	0.9 (0.3–3.1)
High school degree or less				
Events/N	17/122	52/425	68/569	7/62
Model 1: Crude logistic	1.0 (Reference)	0.9 (0.5–1.6)	0.8 (0.5–1.5)	0.8 (0.3–2.0)
Model 2: Unadjusted GEE	1.0 (Reference)	0.8 (0.5–1.5)	0.8 (0.5–1.4)	0.8 (0.3–2.1)
Model 3: Adjusted GEE [a]	1.0 (Reference)	1.0 (0.5–1.8)	1.0 (0.5–1.8)	1.2 (0.4–3.0)

[a] Models adjusted for maternal age at birth (≤21, 22–25, 26–29, ≥30 years), maternal education at time of birth (high school diploma or less, some college, 4-year college grad or higher), and participant race (white, other), sex, year of birth (1969–1974, 1975–1980, 1981–1983), and combined gestational age/birthweight (preterm or <2500 g, term and ≥2500 g); [b] Assigned to a special class because of academic or behavioral problems; Abbreviations: ADD: attention deficit disorder; ADHD, attention deficit hyperactivity disorder; CI, confidence interval; GEE, generalized estimating equation; OR, odds ratio.

References

1. U.S. Food and Drug Administration. Fish: What Pregnant Women and Parents Should Know. Draft Updated Advice by the FDA and EPA. Available online: http://www.fda.gov/Food/FoodborneIllness Contaminants/Metals/ucm393070.htm (accessed on 18 June 2014).
2. U.S. Food and Drug Administration. *A Quantitative Assessment of the Net Effects on Fetal Neurodevelopment from Eating Commercial Fish (As Measured by IQ and also Early Age Verbal Development in Children)*; Center for Food Safety and Applied Nutrition: Silver Spring, MD, USA, 2014.

© 2016 by the authors; licensee MDPI, Basel, Switzerland. This article is an open access article distributed under the terms and conditions of the Creative Commons by Attribution (CC-BY) license (http://creativecommons.org/licenses/by/4.0/).
